# 培门冬酶/左旋门冬酰胺酶治疗结外NK/T细胞淋巴瘤的预后因素分析：多中心回顾性研究

**DOI:** 10.3760/cma.j.issn.0253-2727.2023.08.005

**Published:** 2023-08

**Authors:** 子园 沈, 玺丞 陈, 慧蓉 单, 韬 贾, 伟英 顾, 飞 王, 清良 滕, 玲 王, 春玲 王, 玉叶 史, 颢 张, 雨青 苗, 太岗 朱, 春岩 纪, 静静 叶, 明智 张, 旭东 张, 亮 王, 开林 徐, 威 桑

**Affiliations:** 1 徐州医科大学附属医院血液科，徐州 221002 Department of Hematology, the Affiliated Hospital of Xuzhou Medical University, Xuzhou 221002, China; 2 安徽医科大学公共卫生学院流行病与卫生统计学系，合肥 230032 Department of Epidemiology and Biostatistics, School of Public Health, Anhui Medical University, Hefei 230032, China; 3 宿迁市沭阳县中医院血液科，宿迁 223600 Department of Hematology, Shuyang Hospital of Traditional Chinese Medicine, Suqian 223600, China; 4 连云港第一人民医院血液科，连云港 222002 Department of Hematology, the First People's Hospital of Lianyungang, Lianyungang 222002, China; 5 常州第一人民医院血液科，常州 213003 Department of Hematology, the First People's Hospital of Changzhou, Changzhou 213003, China; 6 泰安市中心医院血液科，泰安 271000 Department of Hematology, Taian Central Hospital, Taian 271000, China; 7 淮安市第一人民医院血液科，淮安 223000 Department of Hematology, Huai'an First People's Hospital, Huaian 223000, China; 8 济宁医学院附属医院血液科，济宁 272000 Department of Hematology, the Affiliated Hospital of Jining Medical University, Jining 272000, China; 9 盐城市第一人民医院血液科，盐城 224001 Department of Hematology, Yancheng First People's Hospital, Yancheng 224001, China; 10 安徽皖北煤电集团总医院血液科，宿州 234000 Department of Hematology, the General Hospital of Wanbei Coal-Electric Group, Suzhou 234000, China; 11 山东大学齐鲁医院血液科，济南 250012 Department of Hematology, Qilu Hospital of Shandong University, Jinan 250012, China; 12 郑州大学第一附属医院肿瘤科，郑州 450052 Department of Hematology, the First Affiliated Hospital of Zhengzhou University, Zhengzhou 450052, China; 13 首都医科大学附属北京同仁医院血液科，北京 100730 Department of Hematology, Beijing Tongren Hospital, Capital Medical University, Beijing 100730, China

**Keywords:** 淋巴瘤，结外NK-T细胞, 预后, 培门冬酶, 天冬酰胺酶, Lymphoma, extranodal NK-T-cell, Prognosis, Pegaspargase, Asparaginase

## Abstract

**目的:**

探索培门冬酶/左旋门冬酰胺酶治疗结外NK/T细胞淋巴瘤（ENKTL）的预后影响因素。

**方法:**

回顾性分析自2014年3月至2021年4月在淮海淋巴瘤协作组11所医疗中心确诊的656例ENKTL患者的临床资料，将患者按照7∶3随机分为训练集（460例）和验证集（196例）两个队列，分析患者的预后影响因素。建立预后评分系统，比较不同模型的预测性能。

**结果:**

患者的中位年龄为46（34，57）岁，男456例（69.5％），鼻部受累561例（85.5％）。203例（30.9％）患者采用了以左旋门冬酰胺酶联合蒽环类药物为基础的化疗方案，接受P-GEMOX（培门冬酶+吉西他滨+奥沙利铂）方案治疗患者的5年总生存率优于接受SMILE（甲氨蝶呤+地塞米松+异环磷酰胺+左旋门冬酰胺酶+依托泊苷）方案的患者（85.9％对63.8％，*P*＝0.004）。多因素分析结果显示性别、CA分期、美国东部肿瘤协作组体能状况（ECOG PS）评分、HGB、EB病毒DNA是ENKTL患者预后的独立影响因素（*P*值均<0.05）。基于本研究筛选出的预后因素的预测性能优于国际预后指数、韩国预后指数和自然杀伤淋巴瘤预后指数。

**结论:**

性别、CA分期、ECOG PS评分、HGB和EB病毒DNA是接受培门冬酶/左旋门冬酰胺酶治疗的ENKTL患者的预后影响因素。

## Abstract (en)

**Objective:**

To explore the prognostic factors of extracellular NK/T cell lymphoma（ENKTL）treated with pegaspargase/L-asparaginase.

**Methods:**

The clinical data of 656 ENKTL patients diagnosed at 11 medical centers in the Huaihai Lymphoma Working Group from March 2014 to April 2021 were retrospectively analyzed. The patients were randomly divided into two groups: a training set（460 cases）and a validation set（196 cases）at 7∶3, and the prognostic factors of the patients were analyzed. A prognostic scoring system was established, and the predictive performance of different models was compared.

**Results:**

Patients' median age was 46（34, 57）years, with 456 males（69.5％）and 561 nasal involvement（85.5％）. 203 patients（30.9％）received a chemotherapy regimen based on L-asparaginase combined with anthracyclines, and the 5-year overall survival rate of patients treated with P-GEMOX regimen（pegaspargase+gemcitabine+oxaliplatin）was better than those treated with SMILE regimen（methotrexate+dexamethasone+cyclophosphamide+L-asparaginase+etoposide）（85.9％ *vs* 63.8％; *P*＝0.004）. The results of multivariate analysis showed that gender, CA stage, the Eastern Cooperative Oncology Group performance status（ECOG PS）score, HGB, and EB virus DNA were independent influencing factors for the prognosis of ENKTL patients（*P*<0.05）. In this study, the predictive performance of the prognostic factors is superior to the international prognostic index, Korean prognostic index, and prognostic index of natural killer lymphoma.

**Conclusion:**

Gender, CA stage, ECOG PS score, HGB, and EB virus DNA are prognostic factors for ENKTL patients treated with pegaspargase/L-asparaginase.

结外NK/T细胞淋巴瘤（ENKTL）是一种罕见且具有高度异质性的非霍奇金淋巴瘤亚型，与EB病毒（EBV）感染有关[1]–[2]。约80％的患者病变位于鼻腔及鼻咽等部位，发病人群主要分布于东亚和南美地区[3]–[4]。由于ENKTL患者具有较高的EBV载量且对蒽环类化疗药物具有耐药性，大多数患者临床预后不佳[5]–[6]。以培门冬酶及左旋门冬酰胺酶为基础的化疗方案极大地改善了患者的预后，但仍有患者出现疾病进展或复发[7]–[8]。因此，早期识别预后不良的患者且优化这些患者的治疗策略显得尤为重要。目前临床上用于评估患者预后的模型主要包括国际预后指数（IPI）、韩国预后指数（KPI）和自然杀伤淋巴瘤预后指数（PINK）[9]–[11]。在以培门冬酶/左旋门冬酰胺酶为基础的联合治疗方案的背景下，预后模型值得进一步探索。本研究旨在通过分析淮海淋巴瘤协作组多中心较大样本的临床数据，探索接受培门冬酶/左旋门冬酰胺酶治疗的ENKTL患者的预后影响因素，进一步指导ENKTL患者的个体化治疗。

## 病例与方法

1. 病例：回顾性收集2014年3月至2021年4月淮海淋巴瘤协作组中11所医疗中心656例ENKTL患者的临床资料。所有ENKTL患者的病理诊断均至少经过两个病理专家确定，符合2016年世界卫生组织造血与淋巴组织恶性肿瘤病理与遗传学分类及诊断标准[12]。缺少临床数据和随访信息以及合并其他实体肿瘤的患者被排除在外。

2. 治疗方案：656例ENKTL患者均接受以培门冬酶/左旋门冬酰胺酶为基础的联合化疗，按照具体方案，分为如下六组，具体为：34例接受SMILE方案（甲氨蝶呤+地塞米松+异环磷酰胺+左旋门冬酰胺酶+依托泊苷），103例接受DDGP方案（地塞米松+顺铂+吉西他滨+培门冬酶）；82例接受P-GEMOX方案（培门冬酶+吉西他滨+奥沙利铂）；203例患者接受以左旋门冬酰胺酶联合蒽环类药物为基础的化疗方案［CHOP-L（环磷酰胺+阿霉素+长春新碱+泼尼松+左旋门冬酰胺酶）、VDLP（长春新碱+柔红霉素+左旋门冬酰胺酶+泼尼松）、EPOCHL（依托泊苷+吡柔比星+长春地辛+环磷酰胺+泼尼松+培门冬酶）］；147例接受以吉西他滨和左旋门冬酰胺酶为基础的化疗方案［GELOX方案（吉西他滨+奥沙利铂+左旋门冬酰胺酶）、GLIDE方案（吉西他滨+左旋门冬酰胺酶+异环磷酰胺+地塞米松+依托泊苷）］；87例接受以非蒽环类药物和左旋门冬酰胺酶为基础的化疗方案［LOP方案（左旋门冬酰胺酶+长春新碱+地塞米松）、LMED方案（甲氨蝶呤+地塞米松+依托泊苷+左旋门冬酰胺酶/培门冬酶）、SVILE方案（甲氨蝶呤+地塞米松+异环磷酰胺+左旋门冬酰胺酶+依托泊苷）］。

3. 预后因素：筛选符合诊断的ENKTL患者，收集患者的临床资料，包括年龄、性别、美国东部肿瘤协作组体能状况评分（ECOG PS评分）、B症状、EBV编码的小RNA、骨髓受累情况、乳酸脱氢酶（LDH）、血肌酐（Cr）、总胆固醇（TC）、甘油三酯（TG）、高密度脂蛋白（HDL）、低密度脂蛋白（LDL）、白蛋白（ALB）、球蛋白（GLB）、WBC、淋巴细胞计数（LYC）、单核细胞（MONO）、HGB、纤维蛋白原（FIB）。所有患者均有完整的IPI、KPI、PINK资料。患者的临床分期采用Ann Arbor分期和CA分期［中国南方肿瘤临床研究协会（CSWOG）和亚洲淋巴瘤协作组（ALSG）分期系统，简称CA分期］进行评估[13]–[14]。

4. 随访：通过查阅患者电子病历系统及纸质病历记录确认患者住院治疗情况，对患者进行电话随访，随访时间截至2022年10月。总生存（OS）期定义为自患者确诊至因任何原因死亡或随访截止的时间间隔。

5. 统计学处理：将满足条件的患者按照7∶3的比例随机划分为训练集和验证集。连续变量采用中位数（*Q*_1_，*Q*_3_）表示，分类变量以例数（百分比）表示；连续变量组间比较采用Mann-Whitney *U*检验，分类变量组间比较采用Pearson *χ*^2^检验；采用Cox比例风险模型进行单因素、多因素分析；采用Kaplan-Meier法绘制患者的生存曲线，组间比较使用Log-rank检验；采用限制立方样条（RCS）确定连续变量的最佳截断值，计算训练集和验证集的C-index，采用ROC曲线进行比较。*P*<0.05为差异有统计学意义，所有统计学分析采用R软件。

## 结果

1. 患者的基线特征：656例ENKTL患者的中位年龄为46（34，57）岁，其中男456例（69.5％）。根据病变累及部位，561例患者为鼻部受累（85.5％）。Ann Arbor Ⅰ/Ⅱ期和CA分期Ⅰ/Ⅱ期的患者分别为509例（77.6％）和463例（63.1％）。截至随访结束，患者的5年OS率为62.2％，中位随访时间为62.8（95％*CI* 59.7～65.8）个月。*χ*^2^检验和Mann-Whitney *U*检验表明训练集和验证集基线临床特征的差异均无统计学意义（*P*值均>0.05），两组的基线临床特征见表1。

**表1:** 训练集与验证集结外NK/T细胞淋巴瘤患者的基线临床特征

临床特征	训练集（460例）	验证集（196例）	*χ*^2^/*Z*值	*P*值
年龄[*M*（*Q*_1_，*Q*_3_）]	46（34，56）	48（35，57）	−0.755	0.450
性别[例（％）]			0.258	0.611
男	323（70.2）	133（67.9）		
女	137（29.8）	63（32.1）		
B症状[例（％）]			3.390	0.066
无	249（54.1）	90（45.9）		
有	211（45.9）	106（54.1）		
ECOG PS评分[例（％）]			0.179	0.672
<2	305（66.3）	134（68.4）		
≥2	155（33.7）	62（31.6）		
原发部位[例（％）]			1.538	0.215
鼻型	399（86.7）	162（82.7）		
非鼻型	61（13.3）	34（17.3）		
Ann Arbor分期[例（％）]			2.257	0.521
Ⅰ	246（53.5）	110（56.1）		
Ⅱ	106（23.0）	47（24.0）		
Ⅲ	23（5.0）	5（2.6）		
Ⅳ	85（18.5）	34（17.3）		
CA分期[例（％）]			1.249	0.741
Ⅰ	121（26.3）	57（29.1）		
Ⅱ	206（44.8）	79（40.3）		
Ⅲ	45（9.8）	19（9.7）		
Ⅳ	88（19.1）	41（20.9）		
KPI[例（％）]			5.955	0.114
低危	147（32.0）	48（24.5）		
低中危	173（37.6）	71（36.2）		
中高危	87（18.9）	49（25.0）		
高危	53（11.5）	28（14.3）		
IPI[例（％）]			1.368	0.713
低危	282（61.3）	117（59.7）		
低中危	96（20.9）	38（19.4）		
中高危	53（11.5）	24（12.2）		
高危	29（6.3）	17（8.7）		
PINK[例（％）]			0.828	0.661
低危	205（44.6）	80（40.8）		
中危	129（28.0）	60（30.6）		
高危	126（27.4）	56（28.6）		

**注** ECOG PS评分：美国东部肿瘤协作组体能状况评分；KPI：韩国预后指数；IPI：国际预后指数；PINK：自然杀伤淋巴瘤预后指数

2. 治疗方案：203例（30.9％）患者采用以左旋门冬酰胺酶联合蒽环类药物为基础的化疗方案，147例（22.4％）采用以吉西他滨和左旋门冬酰胺酶为基础的方案，82例（12.5％）应用DDGP方案，34例（5.2％）应用SMILE方案。应用P-GEMOX方案患者的OS率优于应用SMILE方案的患者（85.9％对63.8％，*P*＝0.004）（图1）。

**图1:**
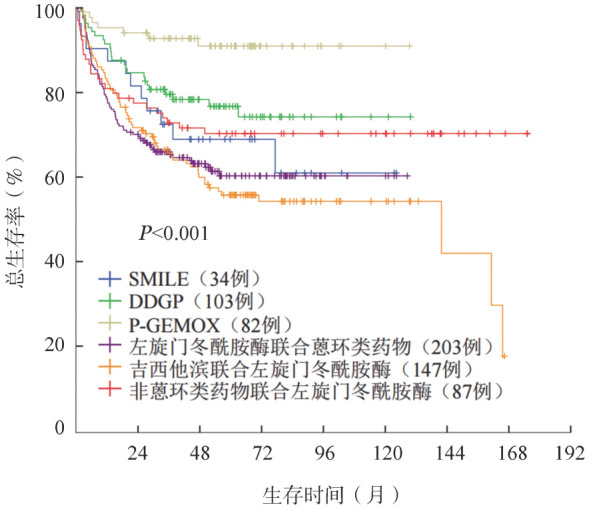
应用不同方案的结外NK/T细胞淋巴瘤患者的总生存曲线

3. 生存分析：单因素分析结果显示，白蛋白、β_2_-微球蛋白、B症状、ECOG PS评分等是ENKTL患者预后的影响因素（*P*值均<0.05）（表2）。多因素分析表明，ECOG PS评分、HGB、性别、EBV DNA和CA分期是患者预后的独立影响因素（表2）。

**表2:** 结外NK/T细胞淋巴瘤患者预后的单因素、多因素分析

因素	单因素分析		多因素分析	
	*HR*（95%*CI*）	*P*值	*HR*（95%*CI*）	*P*值
ECOG PS评分（≥2，<2）	3.546（2.620～4.799）	<0.001	2.721（1.979～3.742）	<0.001
受累部位（鼻外，鼻部）	2.865（2.019～4.065）	<0.001	–	–
HGB	0.971（0.964～0.979）	<0.001	0.985（0.977～0.993）	<0.001
性别（女，男）	0.665（0.469～0.941）	0.021	0.620（0.429～0.895）	0.011
白蛋白	0.928（0.907～0.949）	<0.001	0.972（0.945～1.000）	0.052
EB病毒DNA（阳性，阴性）	2.795（1.954～3.998）	<0.001	2.119（1.449～3.100）	<0.001
骨髓受累（是，否）	3.859（2.389～6.234）	<0.001	1.610（0.942～2.752）	0.082
Ann Arbor分期（Ⅲ/Ⅳ，Ⅰ/Ⅱ）	2.201（1.604～3.022）	<0.001	–	–
B症状（有，无）	2.027（1.494～2.752）	<0.001	–	–
CA分期（Ⅲ/Ⅳ，Ⅰ/Ⅱ）	2.017（1.481～2.747）	<0.001	1.983（1.434～2.741）	<0.001
LDH（≥240 U/L，<240 U/L）	1.969（1.444～2.684）	<0.001	–	–
血肌酐	1.005（1.001～1.010）	0.010	–	–
总胆固醇	0.738（0.634～0.858）	<0.001	0.875（0.752～1.019）	0.087
β_2_-微球蛋白	1.277（1.203～1.356）	<0.001	1.066（0.980～1.160）	0.138

**注** EOCG PS评分：美国东部肿瘤协作组体能状况评分；–：无数据

4. 预后评分系统的建立及验证：使用RCS模型模拟HGB与ENKTL患者预后间的关系，计算出HGB的最佳截断值为131 g/L。构建接受培门冬酶/左旋门冬酰胺酶治疗的ENKTL患者的预后评分系统，每个变量的得分见表3。

**表3:** 接受培门冬酶/左旋门冬酰胺酶治疗的结外NK/T细胞淋巴瘤患者的预后评分系统

预后因素	评分
ECOG PS评分≥2	2
HGB<131 g/L	1
男性	1
EB病毒DNA阳性	2
CA分期Ⅲ/Ⅳ期	1
总分	7

**注** ECOG PS评分：美国东部肿瘤协作组体能状况评分

根据患者在上述预后评分系统中的得分将其分为3组：低危组（0～2分）、中危组（3～5）、高危组（≥6分）。分别绘制训练集和验证集各危险组患者的生存曲线（图2），各组间差异均有统计学意义（*P*值均<0.05）。

**图2:**
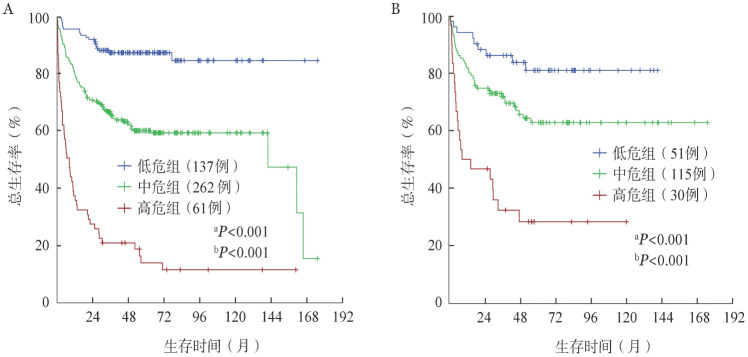
训练集（A）和验证集（B）不同危险组患者的总生存曲线 ^a^*P*为低危组与中危组比较；^b^*P*为中危组与高危组比较

本研究训练集的C-index为0.769（95％*CI* 0.734～0.804），验证集的C-index为0.737（95％*CI* 0.674～0.780）。通过绘制模型的校准图对上述预后评分系统进行内部验证（图3A）。使用验证集的数据对列线图进行外部验证（图3B）。结果显示，校正曲线接近理想曲线，表明预测结果与实际结果具有很好的一致性。

**图3:**
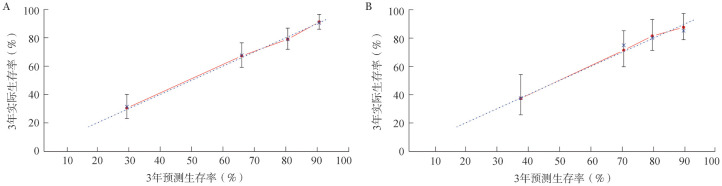
通过训练集（A）及验证集（B）数据对预后评分系统进行验证 红色实线表示预后因素的预测性能，与对角线虚线拟合程度越高，预测效果越好

采用ROC曲线比较上述预后评分系统与KPI、IPI及PINK模型的预测能力。结果提示，在训练集和验证集中，本研究模型的AUC显著高于KPI、IPI和PINK模型（图4）。

**图4:**
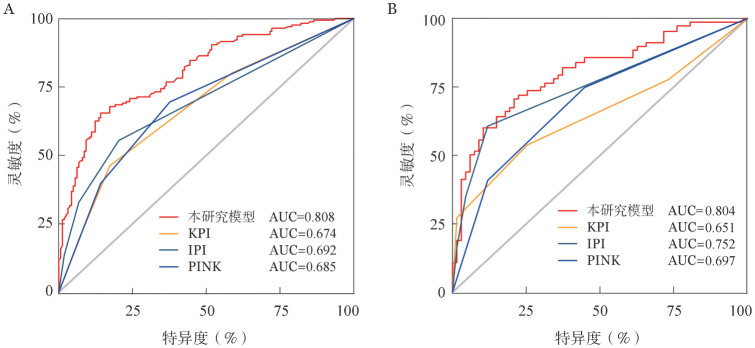
通过训练集（A）和验证集（B）数据比较不同模型预测能力的ROC曲线 KPI：韩国预后指数；IPI：国际预后指数；PINK：自然杀伤淋巴瘤预后指数

## 讨论

ENKTL是一种高度侵袭性的非霍奇金淋巴瘤，由于细胞表达高浓度的多药耐药P-糖蛋白，大多数患者对CHOP或CHOP样化疗方案不敏感[15]。对蒽环类化疗药物的耐药和患者放疗后的高复发率使ENKTL患者的治疗成为难题。随着以培门冬酶/左旋门冬酰胺酶和其他非蒽环类药物为基础的化疗方案的不断应用，ENKTL患者的生存率得到了极大提高，但仍有部分患者复发，尤其是EBV DNA载量高、基因复杂程度高的患者[16]–[17]。在本研究中，我们回顾性分析了淮海淋巴瘤工作组多个医疗中心656例ENKTL患者的临床资料，旨在探索接受培门冬酶/左旋门冬酰胺酶治疗的ENKTL患者的预后影响因素。

在本研究中，基于多中心数据分析，ENKTL患者的5年OS率为62.2％，男性患者居多，且病变部位以鼻腔及鼻咽部为主，上述研究结果与既往研究基本一致[18]。Ann Arbor分期最初为针对霍奇金淋巴瘤的评估系统，但难以对鼻型ENKTL进行准确评估。CA分期是全球第一个系统的ENKTL分期系统。在本研究中，我们分别应用Ann Arbor分期和CA分期评估了患者的临床分期。结果提示，多数患者为疾病早期，多因素分析结果提示CA分期是ENKTL患者的独立预后影响因素，晚期患者常伴有较低的生存率。

本研究将患者的化疗方案分为6类，其中采用以蒽环类药物和左旋门冬酰胺酶为基础化疗方案的患者最多，其次为以吉西他滨和左旋门冬酰胺酶为基础的方案。Lin等[19]证实蒽环类药物联合左旋门冬酰胺酶是初诊ENKTL患者安全且高效的治疗方案，更多有效的治疗方案也值得进一步探索。进一步比较应用不同治疗方案患者的生存情况，结果表明，应用P-GEMOX方案的患者生存率优于应用SMILE方案的患者。SMILE方案通常伴较高的三级血液学不良反应，可能导致感染甚至死亡[20]–[21]。

EBV是ENKTL患者诊断的必要条件，EBV DNA的动态监测有助于评估患者的治疗反应。本研究仅收集到患者的初诊EBV DNA数据，并证明EBV DNA阳性是ENKTL患者的预后不良因素。本研究未能收集到全部患者的EBV DNA动态数据，后续将在前瞻性、大样本研究中进一步证实此结果。

本研究多因素分析提示性别、CA分期、ECOG PS评分、HGB和EBV DNA是ENKTL患者预后的独立影响因素。既往的研究已经表明，低HGB水平与霍奇金淋巴瘤和弥漫大B细胞淋巴瘤的不良预后相关[22]–[23]。值得注意的是，在本研究中，我们发现低HGB水平是ENKTL患者的不良预后因素，也证实了Cao等[24]的研究结果。该研究的训练集和验证集均表明预测结果与实际结果具有较好的一致性。此外，我们还对比了本预后评分系统与ENKTL既往已有预后评分模型（KPI、IPI和PINK评分）的预测准确性。结果提示，与其他模型相比，本研究预后评分系统对ENKTL患者预后的预测准确率更高。

综上，本研究基于多中心ENKTL患者的临床数据进行回顾性分析，在一定程度上减少了既往单中心、小样本研究的偏倚。同时，我们首次探索了ECOG PS评分、EBV DNA、性别、CA分期和HGB水平对患者预后的影响。但由于多中心回顾性数据收集的复杂性，未能收集到所有患者的基因突变情况、动态EBV DNA水平等，需要进一步行前瞻性临床研究加以探索和证实。
